# Effectiveness of the Safe Step Digital Exercise Program to Prevent Falls in Older Community-Dwelling Adults: Randomized Controlled Trial

**DOI:** 10.2196/67539

**Published:** 2025-03-31

**Authors:** Beatrice Pettersson, Lillemor Lundin-Olsson, Dawn A Skelton, Per Liv, Magnus Zingmark, Erik Rosendahl, Marlene Sandlund

**Affiliations:** 1 Department of Community Medicine and Rehabilitation Umeå University Umeå Sweden; 2 School of Health and Life Sciences Glasgow Caledonian University Glasgow United Kingdom; 3 Section of Sustainable Health Department of Public Health and Clinical Medicine Umeå University Umeå Sweden; 4 Health and Social Care Administration Municipality of Östersund Östersund Sweden

**Keywords:** geriatric medicine, aging, accidental falls, independent living, exercise therapy, fall prevention, electronic health, mobile health, preventive medicine, self-management, effectiveness, randomized controlled trial, older adults, digital technology

## Abstract

**Background:**

Falls among older adults are a significant public health issue due to their high incidence, severe consequences, and substantial economic impact. Exercise programs incorporating balance and functional exercises have been shown to reduce fall rates, but adherence and scaling up the interventions remain challenges. Digital technology offers a promising avenue to deliver this type of exercise, potentially improving exercise adherence and enabling self-management of exercise in the aging population.

**Objective:**

This study aims to assess the effectiveness of the Safe Step app, a self-managed, unsupervised, home-based digital exercise program, in reducing fall rates or fall risk in community-dwelling older adults. Additional aims were to describe fall-related injuries in both the exercise and control groups, study attrition, and adherence to the Safe Step exercise program.

**Methods:**

Community-dwelling individuals, aged 70 years or older, who had experienced falls or a decline in balance in the past year were randomized to either an exercise group using the Safe Step app combined with educational videos, or a control group receiving educational videos alone. Both interventions lasted for 1 year. Information regarding fall events was self-reported monthly through questionnaires. Exercise adherence was monitored through questionnaires every third month. Negative binomial and logistic regression estimated the incidence rate ratio of fall rate and the risk ratio (RR) of experiencing falls, respectively. Fall-related injuries, study attrition, and exercise adherence were reported descriptively.

**Results:**

In total, 1628 people were enrolled in the study, 79% were women, and the mean age was 75.8 (SD 4.4) years (range 70-94 years). The intention-to-treat analysis showed no significant difference in fall rates between the exercise and control groups after 12 months (2.21 falls per person-year in the exercise group and 2.41 in the control group; incidence rate ratio 0.92, 95% CI 0.76-1.11; *P*=.37). The risk of experiencing at least 1 fall was significantly lower (11%) in the exercise group compared to the control group (53% vs 59.6%; RR 0.89, 95% CI 0.80-0.99; *P*=.03). No differences were observed regarding the risk of 2 or more falls (34.1% in the exercise group, 37.1% in the control group; RR 0.92, 95% CI 0.79-1.06; *P*=.23). Injurious fall rates were similar between the exercise and control group. During the trial, 161 (20%) participants from the exercise group and 63 (8%) from the control group formally withdrew. The proportion of exercise group participants meeting the 90-minute weekly exercise goal was 12.7%, 13.4%, 8.6%, and 9.1% at 3, 6, 9, and 12 months, respectively.

**Conclusions:**

Access to a self-managed unsupervised digital exercise program can be an effective component of a primary fall prevention strategy for community-dwelling older adults. Further research is needed to explore the mediating factors that influence the outcomes and develop strategies that enhance adherence for optimal impact in this population.

**Trial Registration:**

ClinicalTrials.gov NCT03963570; https://clinicaltrials.gov/study/NCT03963570

**International Registered Report Identifier (IRRID):**

RR2-10.1136/bmjopen-2019-036194

## Introduction

Falls among older adults are acknowledged as a critical public health concern due to the high incidence and substantial economic impact [1]. Falls can result in severe consequences to the individual, such as loss of functional independence and decreased quality of life [2,3], being one of the leading causes of morbidity and mortality in older adults [4]. Globally, the health care costs related to falls among older community-living adults are substantial [5] and are expected to grow with a population that continues to expand [6]. In Sweden, falls are estimated to cost US $1.7 billion in 2020 [7], underscoring the urgent need for cost-effective interventions.

It is well-established that falls among community-dwelling older adults can be prevented with targeted and individualized exercise programs [8]. Incorporating balance and functional exercises into exercise programs for community-dwelling older adults has been estimated to reduce the rate of falls by a quarter. This rate may be further reduced by a third if adding strength exercises to the program [8,9]. The World Health Organization (WHO) has recommended that older adults should perform exercise programs consisting of strength and balance exercises at least 3 days per week to retain functional capacity and prevent falls [1]. Exercise is also recommended as both a primary and secondary prevention measure to prevent falls according to world guidelines for falls prevention [10]. However, fall prevention exercise programs often struggle with low levels of adherence over time [11]. Personal support has been shown to be a facilitator of the continuation of physical activity and fall prevention exercise programs among older adults [12,13]. Nonetheless, personal support can be associated with high costs and is not possible to provide for all older adults at risk [13]. Therefore, there is a pressing need to develop scalable and effective interventions for fall prevention that promote autonomy and independence and support self-management of exercise in the aging population.

Digital technology has increasingly been used to enhance the reach of health-promoting interventions and strengthen the individual’s control over the intervention by being able to take their own initiatives and self-monitor health outcomes. Recent studies indicate that digital technology can be an effective alternative to deliver fall prevention exercise programs to older adults [14-16]. The studies differ in terms of modes of delivery (in a training facility or home-based, supervised or unsupervised), methodology (exergames, virtual reality, mobile apps), as well as time to follow-up (from 10 days to 2 years), which limits comparison [14,15]. However, despite the heterogeneity of the interventions, results of meta-analyses indicate that digital exercise programs can improve both static and dynamic balance [16], lower extremity strength, functional capacity, and health-related quality of life [15] in community-dwelling older adults. In addition, the results suggest that digital exercise programs may have a positive effect on falls [15,16].

Adherence in the short to medium term tends to be higher in digital interventions than in nondigitally delivered interventions but still drops after 12 weeks [17]. The feasibility of home-based digital fall prevention exercise programs is further supported by qualitative studies, where older adults have expressed an appreciation of the flexibility of being able to exercise at preferable times and locations and have access to other supportive strategies not provided in paper-based programs, such as being able to see the exercises in video format [18,19]. However, the evidence for home-based unsupervised digital exercise interventions remains limited [17] and more fall prevention exercise studies with longer intervention periods delivered in a real-life context have been called for supporting implementation [8].

Safe Step is an exercise program delivered via a mobile app to support older adults to exercise in their own homes to prevent falls. The app has been cocreated with older adults to enhance usability as well as ensure motivating support [20,21] and it has been developed to be completely self-managed. As such, older adults can create their own exercise program and monitor progress with support from the app. The app has previously been found acceptable and feasible [22,23]. The primary aim of this randomized controlled trial was to evaluate the effectiveness of the Safe Step app in reducing fall rates and fall risk over a 1-year intervention period. Additional aims were to describe fall-related injuries in both the exercise and control groups, study attrition, and adherence to the Safe Step exercise program.

## Methods

### Ethical Considerations

Ethical approval for this study was obtained from the Swedish Regional Ethical Review Board (reference number 2018/433-31) and the trial was registered at ClinicalTrials.gov (NCT03963570). All participants gave their informed consent to take part in the study by providing their email addresses on the project website after accessing information about the study there. An email was automatically sent to confirm their registration and verify their email address. All data were deidentified. This report does not contain any identifying information or direct quotes. The participants did not receive any financial compensation for their participation.

### Study Design

This randomized, controlled, parallel 2-arm trial evaluated the effectiveness of the Safe Step app (version 2) in combination with monthly educational videos (ie, exercise group), compared to the use of educational videos alone (ie, control group). A detailed protocol has been published [24]. Reporting is aligned with the CONSORT (Consolidated Standards of Reporting Trials) statement [25].

### Participants

Participants were recruited nationwide in Sweden through advertisements on social media, as well as newspapers and senior citizen organizations, between September 2019 and April 2021.

Inclusion criteria were (1) participants aged 70 years or older, (2) having fallen or experienced a decline in perceived postural balance during the past year, (3) having access to a smartphone or tablet and using it regularly, (4) having own email address and using it, (5) being able to understand verbal and written instructions in Swedish, (6) being able to rise from a standard height chair without a person helping, and (7) being able to walk independently without a walking aid indoors. Exclusion criteria were (1) participants having a progressive disease where there is likely to be a decline in strength or balance over the next year, (2) having perceived memory dysfunction that affects everyday life activities, and (3) taking part in strenuous physical exercise (such as dance, aerobics, strength training, running, and skiing) for more than 3 hours a week.

Recruitment and reach have been described in detail elsewhere. In summary, the majority of the participants (76%) were recruited through social media [26]. Potential participants were referred to a study website with information about the study and eligibility criteria. Those interested in taking part could then provide their informed consent by registering their e-mail address through the website and starting the automated recruitment and randomization process.

### Randomization

A permuted block randomization scheme with randomly varied block sizes was generated using the randomizeR R package (R Project) [27] to allocate participants (1:1) to either the exercise or control group. The allocation, according to the generated randomization scheme, was automatically processed by a computer program developed in FLOW, Microsoft Office 365 software after the participants had answered the baseline questionnaire.

### Procedures

After allocation, participants received an email that provided information regarding their allocated intervention. They also received a printable calendar and were encouraged to note any falls (with a definition given) that might occur during the intervention period. Participants in the exercise group received personal log-in details and instructions to download the free Safe Step app (version 2, Apple or Android), which enabled use through smartphones or tablets. If the exercise group participants did not download the app, they received one reminder. When logging into the Safe Step app for the first time, participants viewed an instructional video about how to create and manage their exercise program and an introduction to some of the features of the app.

The participants in the exercise group were instructed to create a program of 10 exercises, by choosing one exercise in each of 10 predetermined categories of strength and balance exercises. They were instructed to choose exercises that were challenging but not too hard and to gradually increase the difficulty of the exercises. The participants were recommended to exercise for at least 30 minutes, 3 times per week, and encouraged to implement the exercises into their everyday activities. Alongside the videos of the exercises performed by older adults, the app provided embedded behavior change techniques such as a calendar for scheduling exercises, reminders, data on performed exercises, and feedback messages on the registered sessions provided by a virtual physiotherapist. More information about the Safe Step program can be found in the study protocol [24].

For each month of the Safe Step RCT, participants in both the exercise and control groups received an email with a hyperlink to an educational video about healthy aging and fall prevention ranging between 5 and 19 minutes in length. A new topic was presented each month and included, for example, “What insights does research offer on the prevalence, risk, and prevention of falls?” and “How does vision impact walking?” (Multimedia Appendix 1).

### Data Collection

All assessments were made through self-reported questionnaires that were delivered automatically by e-mail through the custom-made FLOW program. A monthly questionnaire about recent fall events was delivered along with the monthly educational videos. Participants who did not respond to the questionnaire received one reminder e-mail after 1 week. A follow-up questionnaire was administered at 3, 6, 9, and 12 months after the start of the study, including questions on exercise duration and participant-reported outcomes [24]. Participants who did not respond to the questionnaire received 2 reminder e-mails, the first after 1 week and the second after 2 weeks. In the second reminder email, the participants were asked if they wished to formally withdraw from the trial and a short drop-out questionnaire was provided in the same e-mail, if required.

### Outcomes

#### Rate of Falls and Fall Risk

The primary outcome was the rate of falls (falls per person/year) over the 12-month intervention period. In addition to the fall rate, the fall risk, that is, the proportion of participants who fell during the intervention period, was analyzed. Falls were monitored through monthly questionnaires. A fall was defined as an event in which the person comes to rest inadvertently on the ground or floor, regardless of what caused the fall or if an injury was sustained [24].

#### Injurious Falls

As secondary descriptive outcomes, fall-related injuries were included. Fall-related injuries were reported in the monthly fall questionnaire and were defined as needing assistance from medical care. Participants were also asked to describe the nature of the injury based on the response option (head injury, fracture, or other). If a fracture was sustained, they were asked to describe where the injury was located based on the response options (hip, leg, back, arm, or wrist).

#### Attrition

The characteristics of dropouts in the study are based on completed dropout questionnaires, which were administered at the second reminder after missing the follow-up questionnaire.

#### Adherence

In the follow-up questionnaires administered at 3, 6, 9, and 12 months, the participants were asked to report the number of days per week they had exercised with the Safe Step during the last 3 months. The response options ranged from less than 1 day per week to daily exercise. The participants were also asked to make an estimation of the average duration of the exercise sessions. The mean number of exercise minutes per week was calculated. For those who exercised less than one time per week, zero minutes of exercise were reported.

#### Adverse Events

Falls during exercise with the Safe Step app were monitored in a monthly fall questionnaire. If an injurious fall had occurred during an exercise session, a member of the research group e-mailed the participant asking for a short telephone interview regarding the circumstances leading to the fall event.

### Statistical Analysis

Descriptive statistics, mean, SD, range, or proportion, were calculated to describe the characteristics of the exercise and control group.

Analysis of intervention effects was performed by a statistician blinded to group coding. Primary analyses were performed using an intention-to-treat (ITT) approach, including imputation of missing reports of the number of falls. To account for potentially different mechanisms behind the level of missingness, a two-stage multiple imputation by chained equation (MICE) procedure was used. In the first stage, all participants that had responded to 5 or more monthly questionnaires (ie, more than 40%) had missing monthly fall reports imputed and the total number of falls was thereafter calculated. In the second stage, the total number of falls across the whole year was directly imputed for all participants who answered 4 or fewer of the monthly questionnaires. The imputation procedure was performed separately for the 2 groups. In total, 30 imputed datasets were retrieved and analyzed, and the analyses were pooled using Rubin’s rule. Any falls and multiple falls were imputed using a separate procedure. Details on the imputation procedures can be found in section S2 in Multimedia Appendix 1.

The relative rate of falls between groups was estimated using negative binomial regression, with robust Huber-White standard error estimates. The dependent variable was the total number of falls during the 12-month period, and the independent variable was the group while adjusting for age and sex as prespecified in the study protocol [24]. Age was modeled using restricted cubic splines with three knots placed at the 25th, 50th, and 75th percentiles of the study population’s age distribution. The risk ratio (RR) of any falls was analyzed using logistic regression, with a binary indicator for any falls occurring during the 12-month follow-up period, also adjusting for age and sex in the same way as applied in the analysis of fall rate. The confidence interval for the RR was calculated from the logistic model using the delta method and the R package marginal effects [28]. Using the same procedure as for any fall, the RR number of multiple falls was also estimated. All analyses were also performed on unimputed data as complementary analyses, using the same statistical models as for the imputed analyses, under the assumption that the participants were at risk only during months when the monthly fall questionnaire was answered.

Subgroup analyses were performed for sex, age groups (70-80 vs 80-94 years) and self-rated health (dichotomized to poor+fair vs good+very good) in accordance with the study protocol. In addition, not prespecified in the study protocol, analyses were stratified on whether participants at the baseline questionnaire reported any falls or multiple falls in the previous year before entering the study.

Due to insufficient power for inferential statistics injurious falls are presented descriptively without imputations. Attrition, exercise adherence, and adverse events are also presented descriptively.

## Results

### Overview

**Figure 1:**
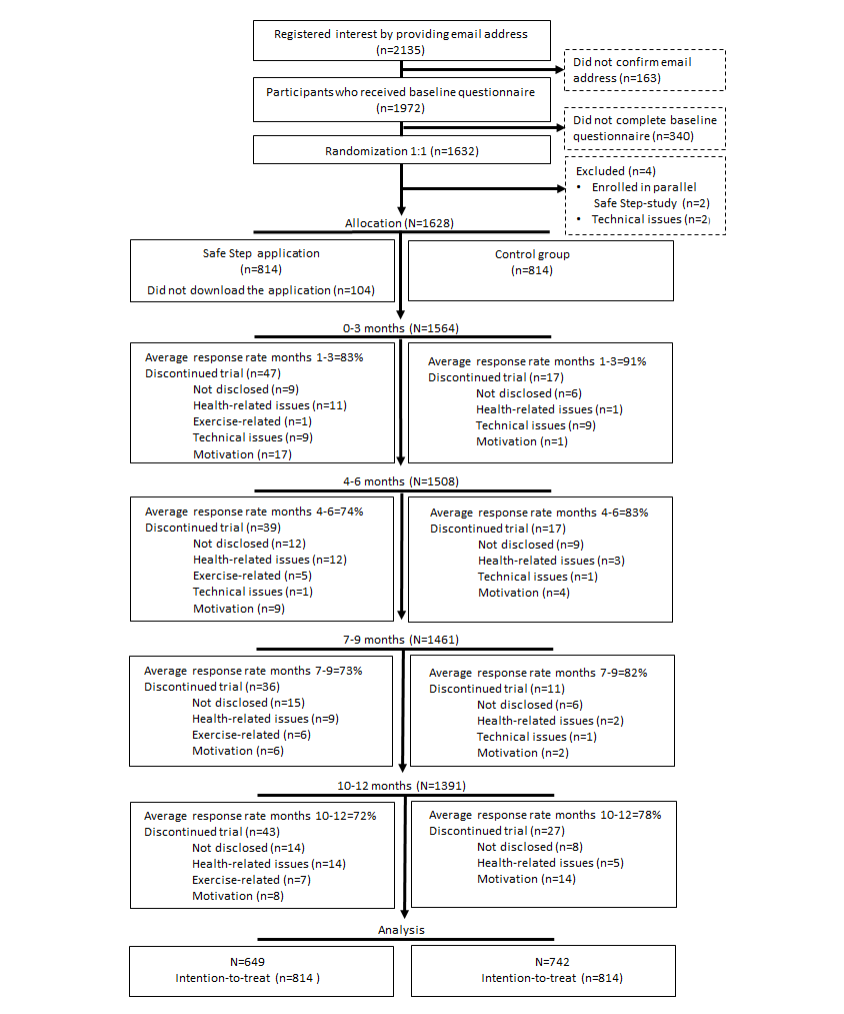
Flowchart of participants throughout the trial. Participants allocated to the exercise intervention who did not download the application (n=104) were not excluded from the trial.

During the recruitment period, 2135 participants registered their interest on the study website. Of these, 1628 participants were enrolled in the study (Figure 1). The mean age was 75.8 (SD 4.4) years (range 70-94 years), and 79% (1292/1628) were women. The majority of the participants had more than 12 years of education (1170/1628, 72%), used smart devices daily (1154/1628, 71%), and approximately one-fifth of the participants (297/1628) used a walking aid. More than 80% (1360/1628) of the participants reported a lack of satisfactory balance, and just over half had sustained at least one fall during the previous year (Table 1).

**Table 1:** Participant characteristics at baseline.

Variable			Exercise (n=814)		Control (n=814)
**Age (years)**					
	Mean (SD)	76.1 (4.5)		75.8 (4.4)	
	Range (min-max)	70-94		70-93	
Women, n (%)			645 (79.2)		647 (79.5)
BMI, mean (SD)			26.7 (4.4)		26.8 (4.4)
**Self-rated overall health, n (%)**					
	Very good or good	423 (52.0)		440 (54.0)	
	Fair	331 (40.7)		336 (41.3)	
	Poor or very poor	60 (7.4)		38 (4.7)	
**Prescription medications/day, n (%)**					
	None	113 (13.9)		122 (15.0)	
	1-3	387 (47.5)		392 (48.2)	
	4 or more	314 (38.6)		300 (36.9)	
Experienced memory dysfunction affecting daily life			28 (3.4)		39 (4.8)
**Education, n (%)**					
	1-9 years	60 (7.4)		63 (7.7)	
	10-12 years	160 (19.7)		175 (21.5)	
	≥12 years	594 (73)		576 (70.8)	
**Use of internet or applications on smart technology, n (%)**					
	Multiple times per day	576 (70.8)		578 (71)	
	Almost every day, or at least once per week	213 (26.2)		216 (26.5)	
	At least once per month but not every week, or more seldom	13 (1.6)		10 (1.3)	
	Never	12 (1.5)		10 (1.2)	
**Residency, n (%)**					
	City	522 (64.1)		529 (65)	
	Town	166 (20.4)		161 (19.8)	
	Village or rural area	126 (15.5)		124 (15.2)	
	Living alone	345 (42.4)		383 (47.1)	
**Falls, n (%)**					
	Falling previous year (n=1602)	451 (55.4)		467 (57.4)	
	Falling ≥2 times previous year (n=1626)	292 (35.9)		298 (36.6)	
**Perceived change in balance, previous year, n (%)**					
	Better	23 (2.9)		39 (4.8)	
	The same	389 (47.8)		398 (48.9)	
	Worse	402 (49.4)		377 (46.3)	
**Perceived balance, n (%)**					
	Very good or good	132 (16.2)		136 (16.7)	
	Fair	407 (50.0)		414 (50.9)	
	Poor or very poor	275 (33.8)		264 (32.5)	
**Perceived leg strength, n (%)**					
	Very good or good	298 (36.6)		286 (35.2)	
	Fair	332 (40.8)		371 (45.6)	
	Poor or very poor	184 (22.6)		157 (19.2)	
	Ability to take brisk 5-min walk	680 (83.5)		708 (87.0)	
Walking aid			155 (19.0)		142 (17.4)
**Physical activity, n (%)**					
	>2 h/week physical daily activities	259 (31.8)		264 (32.4)	
	>2 h/week strenuous physical activities	59 (7.2)		56 (6.9)	

### Falls

In accordance with the ITT analysis, the incidence rate ratio (IRR) indicated no statistical difference in the fall rate between the exercise group and the control group after 12 months (IRR 0.92, 95% CI 0.76-1.11; *P*=.37; Table 2).

The risk of experiencing at least one fall, for example, the proportion of participants who fell during the trial, was 11% lower in the exercise group (RR 0.89, 95% CI 0.80-0.99; *P*=.03). The risk of encountering two or more falls was not statistically different between the groups (RR 0.92, 95% CI 0.79-1.06; *P*=.23; Table 3). Unadjusted and nonimputed data analyses are presented in section S3 in Multimedia Appendix 1.

**Table 2:** Incident rates and incident rate ratios number of falls per person-year during follow-up in the control and exercise group according to intention-to-treat analysis.

Intervention	Number of participants	Total falls	Months, n	IR^a^	IRR^b^ (95% CI)^c^	*P* value
Control	814	1959	9768	2.41	1	—
Exercise	814	1799	9768	2.21	0.92 (0.76-1.11)	.37

^a^IR: incident rate.

^b^IRR: incident rate ratio.

^c^From negative binomial regression analyses, adjusted for age and sex.

**Table 3:** Comparison of exercise versus control group regarding the number of people who experienced one or more falls or two or more falls according to intention-to-treat analysis.

Falls	Exercise (n=814)	Control (n=814)	Risk ratio	95% CI^a^	*P* value
≥1 falls, n (%)^b^	431.1 (53)	485 (59.6)	0.89	0.8-0.99	.03
≥2 falls, n (%)^b^	277.8 (34.1)	302.3 (37.1)	0.92	0.79-1.06	.23

^b^From logistic regression adjusted for age and sex.

^c^While n is an integer in each of the imputed datasets, the decimal arises from pooling the 30 imputed datasets.

### Injurious Falls

A total of 230 injurious falls were reported by 64 individuals. Out of these, 111 occurred in the exercise group and 119 in the control group. (section S4 and Table S4.1 in Multimedia Appendix 1). The most common category of injury constituted “Other injuries” (50%), followed by “Fractures” (29.5%), and finally “Head injuries” (20.5%).

### Subgroup Analyses

Planned subgroup analyses on fall rates and fall risk based on gender, age groups, self-rated health, physical activity, and use of the internet or digital applications did not show any significant mediating effects (see section S5 in Multimedia Appendix 1). In section S6 in Multimedia Appendix 1, subgroup analyses of fall rate and risk of falls are presented, stratified on a history of one or more falls in the previous year. No apparent difference in the intervention effect was observed.

### Attrition

During the trial, 237 (15%) participants formally withdrew from the study by filling in the drop-out questionnaire (Figure 1), 161 (20%) from the exercise group, and 63 (8%) from the control group. Within the control group, participants who had responded to 5 or fewer of the fall questionnaires were more likely to report poorer health-related quality of life, poorer leg strength, and lower levels of physical activity at baseline. No significant differences based on response rates were observed within the exercise group (section S7 and Table S7.1 in Multimedia Appendix 1).

In the exercise group, 104 (13%) participants did not download and activate the Safe Step app. Participants who did not download the app were less prone to being daily users of the internet on their smartphones or tablets, did less daily physical activities, and had a trend toward a worse self-rated overall health at baseline (section S7 and Table S7.2 in Multimedia Appendix 1).

### Adherence

**Figure 2:**
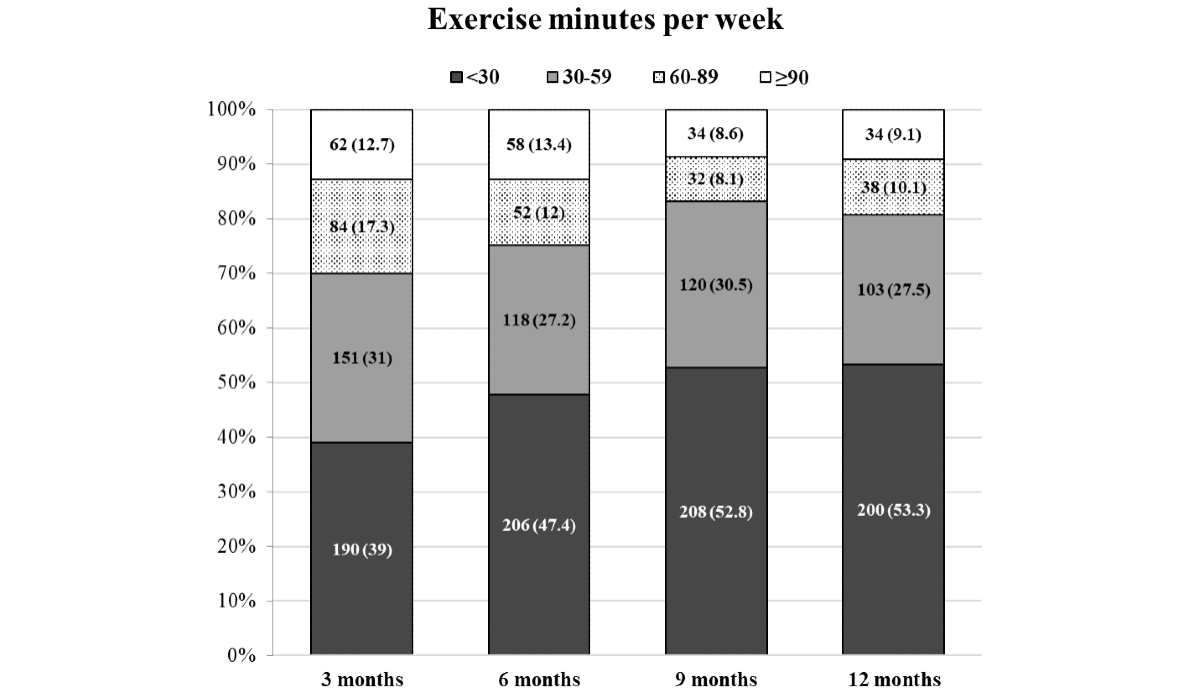
Mean exercise minutes per week at each follow-up based on responders to the follow-up questionnaires. The number in bars refers to the number of participants (percentage).

**Figure 3:**
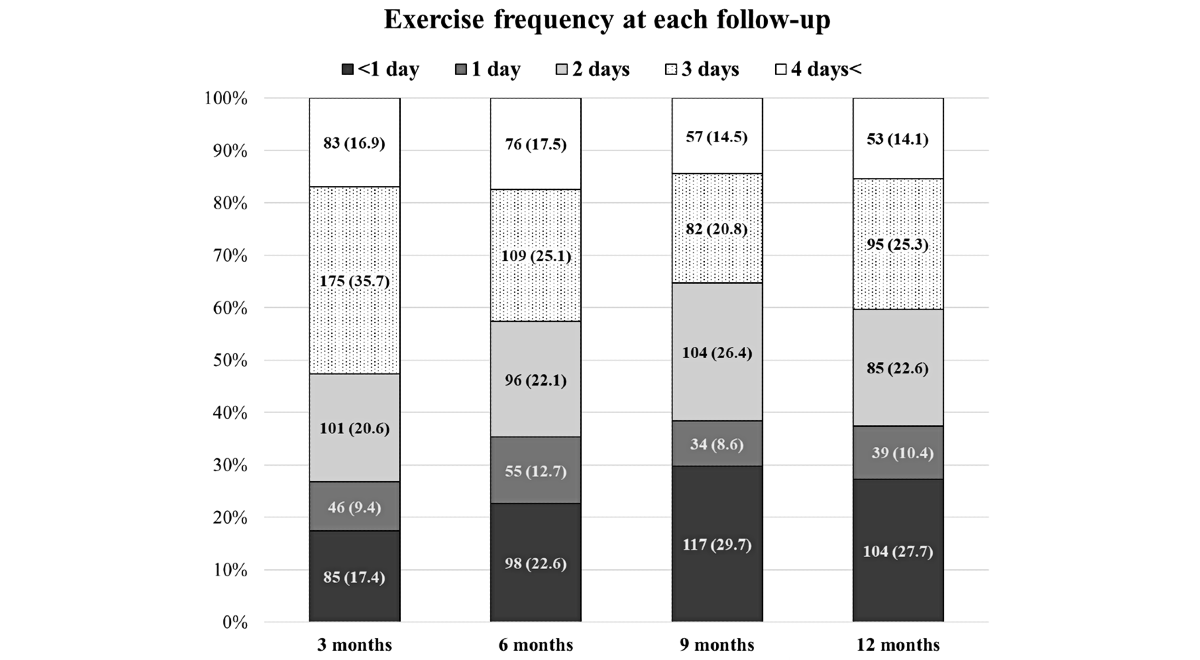
Exercise frequency at each follow-up was based on responders to the follow-up questionnaires. The number in bars refers to the number of participants (percentage).

The proportion of participants in the exercise group who responded to the follow-up questionnaire and who achieved the recommended exercise dose of 90 minutes per week or more at each follow-up was 12.7%, 13.4%, 8.6%, and 9.1%, respectively (Figure 2). Of the participants who answered the questionnaire, the median exercise time per week was 45 (IQR 20-27) minutes at 3 months, 40 (IQR 6-71) minutes at 6 months, 30 (IQR 0-60) minutes at 9 months, and 30 (IQR 0-60) minutes at 12 months. Participants who reported engaging in exercise 3 days or more a week at each follow-up were 52.6%, 42.6%, 35.3%, and 40.4%, respectively (Figure 3).

### Adverse Events

Overall, 38 of 814 (4.7%) participants in the exercise group reported a fall during an exercise session. An actual fall was later confirmed by 5 of 814 participants (0.6%). None of these 5 participants sustained an injury because of falling, but 3 all of them experienced repeated falls during exercise with Safe Step. Further information is available in section S8 in Multimedia Appendix 1.

No negative effects attributed to the exercise were reported by 76%-83% of participants responding to the questionnaire sent out every third month. Joint pain was the most commonly reported negative effect (8%-10.2%), followed by muscle pain (4.3%-6%), dizziness (3%-4.9%), fatigue (1.4%-3.8%), and stress (0.8%-1.6%).

## Discussion

### Principal Findings

The study results show that there was no difference in fall rates between the exercise group and the control group after 12 months. The proportion of individuals who fell at least once was significantly lower in the exercise group, but there was no difference in the proportion of those who fell 2 or more times. In terms of exercise adherence, a small proportion of participants in the exercise group achieved the recommended exercise dose of 90 minutes per week, although 40% (326/814) stated that they exercised with the app for 3 days per week or more at the end of the 12-month period. The study indicates no signs of serious exercise-related adverse events.

### Comparison With Prior Work

The fall rate over 12 months was 8% lower in the exercise group, although the difference was not statistically significant. The effectiveness of the fall rate is lower than reported in previously individually delivered (21%) [8] and digitally delivered (18%) [29] fall prevention exercise programs. However, variations in study designs, populations, and intervention methods must be acknowledged. Unlike prior studies emphasizing personal contact, continuous monitoring, and provision of exercise equipment [29], the evaluation of the Safe Step app prioritized fully self-managed exercise, replicating real-life conditions, while minimizing interactions with the research team. One reason that Safe Step is designed this way is in order to reach large populations, which would not be possible if personal contact was required. The importance of considering the practical implementation and scalability of fall-prevention interventions in real-world settings has previously been highlighted as a need [8]. These considerations are especially important given the challenges observed in community-based physical activity interventions, where effectiveness may diminish when scaled up [30]. This highlights the complexity involved in scaling up interventions and underscores the need for implementation studies within community-based fall prevention programs.

There was a significant reduction (11%) in fall risk in the exercise group. One possible explanation could be that Safe Step, as a fully self-managed intervention, may be best suited for older individuals who have not experienced repeated falls. However, it is worth noting that subgroup analyses were underpowered to confirm this hypothesis, as the study was dimensioned to detect the overall intervention effect. Nonetheless, this observation aligns with recommendations from the World Guidelines for Falls [10]. For individuals with low fall risk, defined according to the World Fall Guidelines as individuals with no falls during the previous year, or one fall without injury and no impaired gait and balance, the guidelines suggest education on fall prevention and advice on physical activity exercise. Individuals with an intermediate risk (one fall without injury the previous year, and gait and balance impairment) are recommended education on fall prevention and tailored exercises. The Safe Step app offers both education on fall prevention and evidence-based exercises focused on balance and functional movements that support daily tasks, tailored by the older adults themselves. However, for individuals with a high risk of falling according to the World Fall Guidelines, additional measures such as multifactorial fall risk assessments and tailored interventions, including exercise are recommended [10]. Thus, for this group, additional measures beyond the scope of Safe Step are recommended. While the guidelines provide a framework, the relationship between fall risk and fall rates remains complex and our results highlight the need for further exploration of mediating factors influencing the effectiveness of exercise in fall prevention. Research by Sherrington et al [9] found consistent effectiveness of exercise interventions across various baseline fall risk levels. However, a review by Wang et al [31] suggests that exercise may be more effective in populations with higher fall rates. Understanding how different populations respond to exercise based on their fall risk profiles can guide the development of targeted interventions.

One plausible explanation for the limited effectiveness of Safe Step in reducing fall rates may be linked to participant engagement with the exercise program. Our study tracked participants’ exercise frequency and duration through regular follow-up questions every 3 months, which can be considered as a relatively crude measure of the actual amount of exercise performed. The results from these assessments revealed that only a modest percentage (9.1%-13.4%) of participants adhered to the recommended exercise duration of 90 minutes per week. Furthermore, both the exercise dose and frequency demonstrated a decline over time, which is a common trend for exercise adherence in fall prevention studies [11]. A factor that may have influenced this decline could be that participants over time no longer needed the app for support as they had memorized the exercises [32]. In addition, the app’s encouragement, provided through tips and feedback messages, suggested integrating training into daily activities [21], which could have complicated participants’ estimations of their training duration. The exercises in the app may also have encouraged additional physical activity, including activities alongside Safe Step exercises, which may have influenced our study results.

It is worth highlighting that 13% of participants randomized to the exercise group never downloaded the app, but their data are still included in the ITT analysis. This nonadoption might be linked to varying levels of comfort and familiarity with digital technologies. This variation in digital proficiency and lack of adherence could indicate that some older adults need more support to initiate and maintain digital exercise programs, but further studies are needed to explore how this kind of support can be arranged [33]. It is noteworthy that this study was conducted between 2019 and 2022, during which the COVID-19 pandemic significantly impacted both individual and societal activities. In a post-RCT interview study, most participants reported that the pandemic influenced their decision to join the study, as many of their regular activities were disrupted due to restrictions [32]. It is plausible that the decline in adherence to the app may be attributed to older individuals returning to their customary physical activities following the removal of social restrictions. The confluence of the study with the pandemic highlights the importance of digital fall prevention training, as it remains unaffected by external factors and can provide stable accessibility.

The small but significant reduction in the risk of experiencing at least one fall not only holds considerable implications for individual well-being but also aligns with cost-effective health care strategies. While the overall results of this digital intervention may be considered modest, the digital nature of the intervention introduces a noteworthy aspect of cost efficiency. In a study by Ambrens et al [34], assessing the cost-effectiveness of the digital exercise application StandingTall, the associated costs for a 2-year intervention fell within the range considered acceptable by decision-makers in Australia. However, a significant portion of these costs was attributed to travel expenses. Interestingly, the intervention costs were reduced by approximately 94% in a sensitivity analysis when the intervention was delivered solely as a telerehabilitation intervention [34]. The scalability and accessibility of a digital application, such as Safe Step, supporting fully self-managed fall prevention exercise make it a relatively inexpensive intervention to administer on a large scale. This economic aspect adds a layer of societal relevance, considering the potential for widespread implementation [7]. Even with a modest reduction in health care utilization related to falls, the cost savings from preventing initial falls can be substantial, which strengthens the case for the integration of digital fall prevention strategies into broader public health initiatives.

### Strengths and Limitations of the Study

This study has several strengths. We included a large sample of community-dwelling adults aged 70 years and older, and our inclusion criteria were broad. Another strength is that the effectiveness of the Safe Step app was tested using a randomized design with limited involvement from the research team during the intervention period. Hence, our results hold strong relevance for real-world implementation. However, it is important to note that the participants in our study were mainly women, highly educated, and technologically proficient, which might limit generalizability.

There are also limitations in our study methods. The way falls were reported may have influenced the outcome. The rate of falls in both groups was high compared to other studies with similar populations [8]. Approximately the same proportion fell during the study as in the year before the study. However, the rate of falls was higher during the intervention period. It is possible that the number of falls is overreported due to difficulties remembering which month they experienced the last fall. It is also plausible that participants recall responding to the months they have fallen but forget the months when they have not. Moreover, the study experienced a high dropout rate, especially in the exercise group, and the reasons behind nonresponses can be multifaceted. There were greater demands on participants in the exercise group, which may have contributed to the higher dropout rate. A control activity with similar commitments could have provided more balance in the results of the dropout rates. Limiting contact with participants exclusively to email communication presented some challenges. For example, one observed difficulty was that participants did not receive emails due to security settings in their email accounts. Therefore, it is plausible that some participants did not receive their allocation information and a portion of the nonresponses to questionnaires may also be attributed to these technical issues rather than a lack of engagement or interest.

### Conclusion

The study indicates that the Safe Step exercise program, a fully self-managed and unsupervised app for fall prevention among community-dwelling older adults, reduces the risk of experiencing any falls, as demonstrated by a significantly lower proportion of participants (11%) in the exercise group who experienced a fall during the intervention period. The results highlight the potential of the Safe Step app as part of a fall prevention strategy for older adults, probably most suitable for those with low to intermediate fall risk. The adherence to the recommended exercise dose was low. However, the results of the study indicate that access to the Safe Step app, supporting full self-management of exercise can reduce the risk of falls, emphasizing its scalability for primary prevention. Further research is needed to explore the mediating factors that influence the outcomes and to develop strategies that enhance adherence for optimal impact in this population.

## Appendix

Multimedia Appendix 1 Supplementary materials.

Multimedia Appendix 2 CONSORT e-Health checklist (V 1.6.1).

## Abbreviations

CONSORT: Consolidated Standards of Reporting Trials
IRR: incidence rate ratio
ITT: intention-to-treat
RR: risk ratio
WHO: World Health Organization

## Data Availability

The datasets generated during and/or analyzed during this study are available from the corresponding author on reasonable request.
