# Supplementary material for: Effectiveness of the Safe Step Digital Exercise Program to Prevent Falls in Older Community-Dwelling Adults: Randomized Controlled Trial
Source: J Med Internet Res. 2025 Mar 31;27:e67539. doi: 10.2196/67539 (PMC11997535; doi:10.2196/67539)
Supplement: Multimedia Appendix 1 [file jmir_v27i1e67539_app1.docx]

**Supplementary materials - Safe Step randomized controlled trial**

Table of Content

[S1. Monthly educational videos 2](#_Toc179806098)

[S2. Imputation procedures 3](#_Toc179806099)

[S3. Supplementary analyses of fall rate and fall risk 5](#_Toc179806100)

[S4. Description of fall related injuries 6](#_Toc179806101)

[S5. Pre-planned subgroup analyses 7](#_Toc179806102)

[Subgroup analyses fall rate 7](#_Toc179806103)

[Subgroup analyses experiencing any falls 9](#_Toc179806104)

[S6. Exploratory subgroup analyses stratified on fall history 11](#_Toc179806105)

[Subgroup analyses fall rate 11](#_Toc179806106)

[Subgroup analyses of experiencing any fall 11](#_Toc179806107)

[S7. Drop-out analysis 12](#_Toc179806108)

[S8. Adverse events during exercise with the Safe Step application 14](#_Toc179806109)

S1. Monthly educational videos

The topics of the monthly educational videos sent out during the Safe Step RCT:

- What insights does research offer on the prevalence, risk and prevention of falls?
- How does the food we eat affect balance?
- Better health from physical activity and exercise
- How does vision impact walking?
- Prescription drugs effect on balance
- Identifying and removing home hazards
- Risk of falling due to pain and dizziness
- Osteoporosis and falls
- How do our feet affect balance and fall risk?
- Can urine incontinence increase the risk of falling?
- Thinking ahead – risk taking, risk awareness and fear of falling
- Your muscles: the body’s pharmacy

S2. Imputation procedures

**Description of imputation procedure for fall rate**

Multiple imputations by chained equations were performed in a two-stage procedure. The following procedure was performed separately for the exercise group and the control group, generating 30 imputed data sets.

***Stage 1***

In a subset of the data, consisting of all participants that had responded to five or more monthly questionnaires, missing monthly fall reports were imputed. Imputations were conditioned on the following variables:

| **Predictor variables** | **Scale** |
| --- | --- |
| Age | Numeric |
| Sex | Men/women |
| Self-rated health | very poor, poor, fair, good, very good |
| Perceived balance | very poor, poor, fair, good, very good |
| prescription medications/day | None; 1, 2, 3, 4 or more |
| self-reported number of falls last year | Numeric |
| Perceived relative walking speed | Much slower, slightly slower, equally fast, slightly faster or much faster compared to other people in same age |
| Perceived leg strength | very poor, poor, fair, good, very good |
| Number of responded monthly questionnaires | Numeric (0-12) |
| Fall rate across the reported monthly fall rate | Numeric (falls/year). |

Imputations were performed using the *mice* function from the R package *mice*. Predictive mean matching from 10 potential donors was used for imputing numeric variables, and polytomous regression imputation for categorical variables.

For each participant in each imputed data set, the total number of falls was computed by summing the monthly fall reports.

***Stage 2***

A variable for the total number of falls was created in data for the subset of participants that had responded to 4 or less monthly questionnaires, and the values were all set as missing. The data set was then merged with the imputed data sets from stage 1. Again, mice were used, now to directly impute the missing total number of falls among the participants with 4 or less monthly fall reports, in a similar procedure as for stage 1.

The R code *TeachingDemos::char2seed("Safe step")* was used to generate the seed. After the procedure, each imputed dataset from the exercise group was merged with a corresponding imputed data set from the control group.

**Description of imputation procedure for any falls**

The following procedure was used, separately for the exercise group and the control group, to generate the 30 imputed data sets for the

A variable “Any falls” was created, defined as “TRUE” for all participants with a reported fall in any of the monthly questionnaires. For participants with no registered falls, the variable “Any falls” was set as FALSE if the number of responded monthly questionnaires was at least 11. For participants with no registered falls and with less than 11 responded monthly questionnaires, faller was set as missing.

Imputations were performed using the *mice* function from the R package *mice,* conditioning on the same variables as for fall rate, except that the variable “Any falls” replaced “Fall rate”. Predictive mean matching from 10 potential donors was used for imputing numeric variables, logistic regression for binary variables, and polytomous regression imputation for categorical variables with more than 2 categories.

The same seed as for the fall rate imputation procedure was used (*TeachingDemos::char2seed("Safe step"))*. After the procedure, each imputed dataset from the exercise group was merged with a corresponding imputed data set from the control group.

# S3. Supplementary analyses of fall rate and fall risk

| **Intervention** |  | **n** | **Total falls** | **nMonths** | **IR** | **IRR [CI]** | **p** |
| --- | --- | --- | --- | --- | --- | --- | --- |
| Control |  | 814 | 1536 | 9768 | 1.889 | 1.00 |  |
| Exercise |  | 814 | 1327 | 9768 | 1.631 | 0.86 [0.72, 1.04] | 0.126 |

Supplementary table S3.1. IRs and IRR of falls per person-year during follow-up in the control and Safe Step intervention group of unadjusted data according to ITT analysis.

Supplementary table S3.2. IRs and IRR of falls per person-year during follow-up in the exercise and control group according to unimputed analyses.

| **Intervention** | **Total falls** | **nMonths** |  | **Unadjusted model** | | |  | **Adjusted model** | | |
| --- | --- | --- | --- | --- | --- | --- | --- | --- | --- | --- |
|  |  |  | **IR** | **IRR** | **95% CI** | **p** |  | **IRR*** | **95% CI** | **p** |
| Control (n=799) | 1494 | 7786 | 2.30 | 1.00 |  |  |  | 1.00 |  |  |
| Exercise (n=744) | 1160 | 6508 | 2.14 | 0.92 | 0.79-1.08 | 0.328 |  | 0.92 | 0.76-1.12 | 0.410 |

*From negative binomial regression analyses with observation months as exposure term and adjusted for age and sex.

Supplementary table S3.3. Comparison of exercise versus control group regarding number of people who experienced one or more falls or two or more falls according to unimputed analyses.

| **Variable** | **Exercise** (n=744) | **Control**  (n=799) | **Unadjusted model** | | |  | **Adjusted model** | | |
| --- | --- | --- | --- | --- | --- | --- | --- | --- | --- |
|  |  |  | **RR** | **95% CI** | **p** |  | **RR*** | **95% CI** | **p** |
| **≥1 falls**, n (%) | 383 (51.5) | 454 (56.8) | 0.91 | 0.83–0.99 | 0.035 |  | 0.90 | 0.82–0.99 | 0.027 |
| **≥2 falls**, n (%) | 236 (31.7) | 278 (34.8) | 0.91 | 0.71–1.05 | 0.201 |  | 0.90 | 0.78–1.04 | 0.148 |

*Adjusted for age and sex.

# S4. Description of fall related injuries

Supplementary table S4.1. Presentation of injurious falls by intervention arm and participants who have responded to at least one fall report.

| **Injurious falls** | **Total** (n=1544) | **Exercise** (n=744) | **Contr**o**l** (n=799) |
| --- | --- | --- | --- |
|  |  |  |  |
| Number of participants sustaining injurious falls, n (%) | 164 (10.6) | 79 (10.6) | 85 (10.6) |
| Number of participants sustaining multiple injurious falls, n (%) | 44 (2.9) | 22 (3.0) | 22 (2.7) |
| Number of injurious falls, n | 230 | 111 | 119 |
| Injurious falls per-person year | 0.19 | 0.20 | 0.18 |
| Number of injuries reported*, n | 254 | 124 | 130 |
| Head injuries | 52 (20.5) | 27 (21.8) | 25 (19.2) |
| Other injuries | 127 (50.0) | 63 (50.8) | 64 (49.2) |
| Fractures | 75 (29.5) | 34 (27.4) | 41 (31.5) |
| Site of fracture*, n (%) |  |  |  |
| Back | 8 (10.7) | 1 (2.9) | 7 (17.1) |
| Hip | 8 (10.7) | 1 (2.9) | 7 (17.1) |
| Leg other | 25 (33.3) | 13 (38.2) | 12 (29.3) |
| Wrist | 15 (20.0) | 7 (20.6) | 8 (19.5) |
| Arm other | 19 (25.3) | 12 (35.3) | 7 (17.1) |

An injurious fall is defined as needing assistance from medical care. Number of participants in group total represent those who have answered at least one fall report. *Note that one person in the control group contributed with a substantial number of injurious falls, the majority of the injuries occurring during the last three months of the intervention period; 3 hip fractures, 1 back fracture, 3 arm fractures other, 2 leg fractures other, and1 head injury.

# S5. Pre-planned subgroup analyses

## **Subgroup analyses fall rate**

***Stratification on sex***

Supplementary table S5.1 Incident rate ratio and confidence intervals for imputed analyses of fall rate, stratified on sex and adjusted for age. When testing for significant interaction between treatment group and sex, p was 0.84.

|  | **Men** | | | |  | **Women** | | | |
| --- | --- | --- | --- | --- | --- | --- | --- | --- | --- |
|  | **n** | **IRR** | **95% CI** | **p** |  | **n** | **IRR** | **95% CI** | **p** |
| Control | 167 | 1.00 |  |  |  | 647 | 1.00 |  |  |
| Exercise | 169 | 0.87 | 0.59-1.30 | 0.509 |  | 645 | 0.93 | 0.59- 1.30 | 0.489 |

***Stratification on age groups***

Supplementary table S5.2 Incident rate ratio and confidence intervals for imputed analyses stratified on age groups and adjusted for sex. When testing for significant interaction between treatment group and age groups, p was 0.65.

|  | **70-79 years old** | | | |  | **80-94 years old** | | | |
| --- | --- | --- | --- | --- | --- | --- | --- | --- | --- |
|  | **n** | **IRR** | **95% CI** | **p** |  | **n** | **IRR** | **95% CI** | **p** |
| Control | 652 | 1.00 |  |  |  | 162 | 1.00 |  |  |
| Exercise | 632 | 0.93 | 0.76-1.15 | 0.513 |  | 182 | 0.87 | 0.62-1.22 | 0.411 |

***Stratification on self-rated health***

Supplementary table S5.3 Incident rate ratio and confidence intervals for imputed analyses stratified on Self rated health and adjusted for sex. When testing for significant interaction between treatment group and self-rated heatlh, p was 0.82.

|  | **Good or very good health** | | | |  | **Fair or poor health** | | | |
| --- | --- | --- | --- | --- | --- | --- | --- | --- | --- |
|  | **n** | **IRR** | **95% CI** | **p** |  | **n** | **IRR** | **95% CI** | **p** |
| Control | 440 | 1.00 |  |  |  | 374 | 1.00 |  |  |
| Exercise | 423 | 0.96 | 0.77-1.19 | 0.683 |  | 391 | 0.88 | 0.67-1.15 | 0.350 |

***Stratification on physical daily activities***

Supplementary table S5.4 Incident rate ratio and confidence intervals for imputed analyses stratified on Self rated health, adjusted for sex and age. When testing for significant interaction between treatment group and physical daily activities, p was 0.18.

|  | **>2 hours/ week physical daily activities** | | | |  | **≤ 2 hours/ week physical daily activities** | | | |
| --- | --- | --- | --- | --- | --- | --- | --- | --- | --- |
|  | **n** | **IRR** | **95% CI** | **p** |  | **n** | **IRR** | **95% CI** | **p** |
| Control | 262 | 1.00 |  |  |  | 537 | 1.00 |  |  |
| Exercise | 232 | 1.11 | 0.79-1.55 | 0.558 |  | 505 | 0.86 | 0.68-1.10 | 0.234 |

***Stratification on daily use of smartphone and tablets***

Supplementary table S5.5 Incident rate ratio and confidence intervals for imputed analyses stratified on daily use of smartphone and tablets, adjusted for sex and age. When testing for significant interaction between treatment group and daily use of smartphone and tablets, p was 0.73.

|  | **Multiple use of smartphone/tablets everyday** | | | |  | **Not multiple use of smartphone/tablets everyday** | | | |
| --- | --- | --- | --- | --- | --- | --- | --- | --- | --- |
|  | **n** | **IRR** | **95% CI** | **p** |  | **n** | **IRR** | **95% CI** | **p** |
| Control | 578 | 1.00 |  |  |  | 236 | 1.00 |  |  |
| Exercise | 576 | 0.82 | 0.66-1.02 | 0.072 |  | 238 | 1.27 | 0.94-1.73 | 0.121 |

**Subgroup analyses experiencing any falls**

***Stratification on sex***

Supplementary table S5.6 Incident rate ratio and confidence intervals for imputed analyses of any falls, stratified on sex and adjusted for age. When testing for significant interaction between treatment group and sex, p was 0.84.

|  | **Men** | | | |  | **Women** | | | |
| --- | --- | --- | --- | --- | --- | --- | --- | --- | --- |
|  | **n** | **RR** | **95% CI** | **p** |  | **n** | **RR** | **95% CI** | **p** |
| Control | 167 | 1.00 |  |  |  | 647 | 1.00 |  |  |
| Exercise | 169 | 0.87 | 0.70-1.07 | 0.188 |  | 645 | 0.89 | 0.80-1.00 | 0.057 |

***Stratification on age groups***

Supplementary table S5.7 Incident rate ratio and confidence intervals for imputed analyses stratified on age groups and adjusted for sex. When testing for significant interaction between treatment group and age groups, p was 0.65.

|  | **70-79 years old** | | | |  | **80-94 years old** | | | |
| --- | --- | --- | --- | --- | --- | --- | --- | --- | --- |
|  | **n** | **RR** | **95% CI** | **p** |  | **n** | **RR** | **95% CI** | **p** |
| Control | 652 | 1.00 |  |  |  | 162 | 1.00 |  |  |
| Exercise | 632 | 0.87 | 0.78-0.98 | 0.025 |  | 182 | 0.96 | 0.77- 1.18 | 0.675 |

***Stratification on self-rated health***

Supplementary table S5.8 Incident rate ratio and confidence intervals for imputed analyses stratified on Self rated health and adjusted for sex. When testing for significant interaction between treatment group and self-rated health, p was 0.15.

|  | **Good or very good health** | | | |  | **Fair or poor health** | | | |
| --- | --- | --- | --- | --- | --- | --- | --- | --- | --- |
|  | **n** | **IRR** | **95% CI** | **p** |  | **n** | **IRR** | **95% CI** | **p** |
| Control | 440 | 1.00 |  |  |  | 374 | 1.00 |  |  |
| Exercise | 423 | 0.87 | 0.74-1.10 | 0.057 |  | 391 | 0.91 | 0.80-1.05 | 0.187 |

***Stratification on physical daily activities***

Supplementary table S5.9 Incident rate ratio and confidence intervals for imputed analyses stratified on >2 hours/ week physical daily activities, adjusted for sex and age. When testing for significant interaction between treatment group and physical daily activities, p was 0.95.

|  | **>2 hours/ week physical daily activities** | | | |  | **≤2 hours/ week physical daily activities** | | | |
| --- | --- | --- | --- | --- | --- | --- | --- | --- | --- |
|  | **n** | **RR** | **95% CI** | **p** |  | **n** | **RR** | **95% CI** | **p** |
| Control | 264 | 1.00 |  |  |  | 550 | 1.00 |  |  |
| Exercise | 259 | 0.91 | 0.70-1.55 | 0.558 |  | 555 | 0.90 | 0.80-1.00 | 0.055 |

***Stratification on daily use of smartphone and tablets***

Supplementary table S5.10 Incident rate ratio and confidence intervals for imputed analyses stratified on daily use of smartphone and tablets, adjusted for sex and stratified on daily use of smartphones or tablet. When testing for significant interaction between treatment group and use of smartphone and tablets, p was 0.28.

|  | **Daily multiple use of smartphone/tablets** | | | |  | **Non-daily multiple use of smartphone/tablets** | | | |
| --- | --- | --- | --- | --- | --- | --- | --- | --- | --- |
|  | **n** | **RR** | **95% CI** | **p** |  | **n** | **RR** | **95% CI** | **p** |
| Control | 578 | 1.00 |  |  |  | 236 | 1.00 |  |  |
| Exercise | 576 | 0.84 | 0.74-0.95 | 0.005 |  | 238 | 1.05 | 0.88-1.25 | 0.629 |

S6. Exploratory subgroup analyses stratified on fall history

## **Subgroup analyses fall rate**

***Stratification on any self-reported falls previous year***

Supplementary table S6.1 Incident rate ratio and confidence interval for imputed analyses of fall rate, stratified on ≥ 1 self-reported fall previous year, Models were adjusted for sex and age. When testing for significant interaction between treatment group and any fall previous year, p was 0.93.

|  | **Reported no falls previous year** | | | |  | **Reported ≥ 1 fall previous year** | | | |
| --- | --- | --- | --- | --- | --- | --- | --- | --- | --- |
|  | **n** | **IRR** | **95% CI** | **p** |  | **n** | **IRR** | **95% CI** | **p** |
| Control | 347 | 1.00 |  |  |  | 467 | 1.00 |  |  |
| Exercise | 367 | 0.93 | 0.69-1.24 | 0.603 |  | 450 | 0.94 | 0.76-1.16 | 0.567 |

***Stratification on more than 1 self-reported fall previous year***

Supplementary table S6.2 Incident rate ratio and confidence interval for imputed analyses of fall rate, stratified on ≥ 2 self-reported fall previous year, Models were adjusted for sex and age. When testing for significant interaction between treatment group and ≥ 2 self-reported fall previous year, p was 0.67.

|  | **Reported <2 falls previous year** | | | |  | **Reported ≥ 2 fall previous year** | | | |
| --- | --- | --- | --- | --- | --- | --- | --- | --- | --- |
|  | **n** | **IRR** | **95% CI** | **p** |  | **n** | **IRR** | **95% CI** | **p** |
| Control | 516 | 1.00 |  |  |  | 298 | 1.00 |  |  |
| Exercise | 522 | 0.97 | 0.77-1.21 | 0.758 |  | 292 | 0.91 | 0.71-1.16 | 0.429 |

## **Subgroup analyses of experiencing any fall**

***Stratification on any self-reported falls previous year***

Supplementary table S6.3 Relative risk (RR)e ratio and confidence intervals for imputed analyses of any falls, stratified on ≥ 1 self-reported fall the previous year. When testing for significant interaction between treatment group and any fall previous year, p was 0.41.

|  | **Reported no falls previous year** | | | |  | **Reported ≥ 1 fall previous year** | | | |
| --- | --- | --- | --- | --- | --- | --- | --- | --- | --- |
|  | **n** | **RR** | **95% CI** | **p** |  | **n** | **RR** | **95% CI** | **p** |
| Control | 347 | 1.00 |  |  |  | 467 | 1.00 |  |  |
| Exercise | 364 | 0.93 | 0.73-1.17 | 0.517 |  | 450 | 0.88 | 0.80-0.97 | 0.014 |

***Stratification on more than 1 self-reported fall previous year***

Supplementary table S6.4 Relative risk (RR)e ratio and confidence intervals for imputed analyses of any falls, stratified on ≥ 2 self-reported fall previous year. When testing for significant interaction between treatment group and ≥ 2 self-reported fall previous year, p was 0.73.

|  | **Reported <2 falls previous year** | | | |  | **Reported ≥ 2 fall previous year** | | | |
| --- | --- | --- | --- | --- | --- | --- | --- | --- | --- |
|  | **n** | **RR** | **95% CI** | **p** |  | **n** | **RR** | **95% CI** | **p** |
| Control | 516 | 1.00 |  |  |  | 298 | 1.00 |  |  |
| Exercise | 522 | 0.86 | 0.73-1.02 | 0.076 |  | 292 | 0.93 | 0.83- 1.03 | 0.163 |

# S7. Drop-out analysis

|  |  | **Safe Step** | |  |  | **Control group** | |  |
| --- | --- | --- | --- | --- | --- | --- | --- | --- |
| **Variable** |  | ≤5 (n=245) | >5  (n=569) | p-value |  | ≤5 (n=124) | >5  (n=690) | p-value |
| **Age**, mean±SD |  | 76.02±4.6 | 76.07±4. | 0.75 |  | 76.02±4.6 | 75.74±4.4 | 0.56 |
| **Women**, n (%) |  | 193 (78.8) | 452 (79.4) | 0.83 |  | 95 (76.6) | 555 (80.4) | 0.11 |
| **Falls previous year**, n (%) |  | 125 (51.0) | 325 (57.1) | 0.10 |  | 75 (60.5) | 392 (56.8) | 0.45 |
| **Prescription medications/day**, n (%) |  |  |  | 0.35 |  |  |  | 0.51 |
| None |  | 32 (13.1) | 81 (14.2) |  |  | 14 (11.3) | 108 (15.7) |  |
| 1–3 |  | 127 (51.8) | 260 (45.7) |  |  | 58 (46.7) | 334 (48.4) |  |
| 4 or more |  | 86 (35.1) | 228 (40.1) |  |  | 52 (42.0) | 248 (35.9) |  |
| **Self-rated overall health**, n (%) |  |  |  | 0.80 |  |  |  | **0.008** |
| Very good or Good |  | 133 (54.3) | 290 (51.0) |  |  | 58 (46.7) | 382 (55.3) |  |
| Fair |  | 93 (37.9) | 238 (41.8) |  |  | 54 (43.5) | 282 (40.9) |  |
| Poor or Very poor |  | 19 (7.8) | 41 (7.2) |  |  | 12 (9.8) | 26 (3.8) |  |
| **Perceived balance**, n (%) |  |  |  | 0.45 |  |  |  | 0.06 |
| Very good or Good |  | 37 (15.1) | 95 (16.7) |  |  | 15 (12.1) | 121 (17.5) |  |
| Fair |  | 134 (54.7) | 273 (48.0) |  |  | 61 (49.2) | 353 (51.2) |  |
| Poor or Very poor |  | 74 (30.2) | 201 (35.3) |  |  | 48 (38.7) | 216 (31.3) |  |
| **Perceived leg strength**, n (%) |  |  |  | 0.70 |  |  |  | **0.004** |
| Very good or Good |  | 89 (36.1) | 209 (36.8) |  |  | 36 (29.0) | 250 (36.2) |  |
| Fair |  | 102 (41.6) | 230 (40.4) |  |  | 51 (41.1) | 320 (46.4) |  |
| Poor or Very poor |  | 54 (22.0) | 130 (22.8) |  |  | 37 (29.9) | 120 (17.4) |  |
| **Physical activity**, n (%) |  |  |  |  |  |  |  |  |
| Physical daily activities (hours/week) |  |  |  | 0.34 |  |  |  | **0.003** |
| <1 |  | 62 (25.3) | 96 (16.8) |  |  | 34 (27.4) | 126 (18.3) |  |
| 1-2 |  | 103 (42.0) | 294 (51.7) |  |  | 61 (49.2) | 329 (47.6) |  |
| >2 |  | 80 (32.7) | 179 (31.5) |  |  | 29 (23.4) | 235 (34.1) |  |
| Strenuous physical activities (hours/week) |  |  |  | 0.76 |  |  |  | 0.92 |
| <1 |  | 142 (58.0) | 326 (57.3) |  |  | 72 (58.0) | 403 (58.5) |  |
| 1-2 |  | 88 (35.9) | 199 (35.0) |  |  | 43 (34.7) | 240 (34.7) |  |
| >2 |  | 15 (6.1) | 44 (7.7) |  |  | 9 (7.3) | 47 (6.8) |  |

Supplementary table S7.1. Analyses of within group differences based on response rate of the monthly fall questionnaires. Analyses are divided based on response of five or less (≤41.7%) or more than five questionnaires (>41.7%) of the twelve questionnaires.

| **Variable** | **Downloaded application**  (n=710) | **No application**  (n=104) | **p-value** |
| --- | --- | --- | --- |
| **Age**, mean±SD (min-max) | 75.98±4.4 (70-90) | 76.79±4.8 (70-94) | 0.09 |
| **Women**, n (%) | 556 (78.3) | 89 (85.6) | 0.09 |
| **Falls previous year**, n (%) | 391 (55.1) | 59 (56.7) | 0.75 |
| **Education**, n (%) |  |  | 0.90 |
| 1–9 years | 53 (7.4) | 7 (6.7) |  |
| 10–12 years | 138 (19.4) | 22 (21.2) |  |
| 12+ years | 519 (73.2) | 75 (72.1) |  |
| **Use of Internet or applications on smart technology, n (%)** |  |  | **<0.001** |
| Multiple times per day | 522 (73.5) | 54 (51.9) |  |
| Almost every day, or at least   once per week | 171 (24.0) | 43 (40.3) |  |
| At least once per month but not  every week, or more seldom | 11 (1.5) | 2 (2.0) |  |
| Never | 6 (0.8) | 6 (5.8) |  |
| **Prescription medications/day**, n (%) |  |  | 0.45 |
| None | 100 (14.1) | 13 (12.5) |  |
| 1–3 | 335 (48.1) | 52 (50.0) |  |
| 4 or more | 275 (38.7) | 39 (37.5) |  |
| **Self-rated overall health**, n (%) |  |  | 0.08 |
| Very good or Good | 375 (52.8) | 48 (46.1) |  |
| Fair | 290 (40.8) | 41 (39.4) |  |
| Poor or Very poor | 45 (6.4) | 15 (14.5) |  |
| **Perceived balance**, n (%) |  |  | 0.33 |
| Very good or Good | 119 (16.8) | 13 (12.5) |  |
| Fair | 354 (49.9) | 53 (50.9) |  |
| Poor or Very poor | 237 (33.3) | 38 (36.5) |  |
| **Perceived leg strength**, n (%) |  |  | 0.50 |
| Very good or Good | 263 (37.0) | 35 (33.6) |  |
| Fair | 288 (40.6) | 44 (42.3) |  |
| Poor or Very poor | 159 (22.4) | 25 (24.1) |  |
| **Physical activity**, n (%) |  |  |  |
| Physical daily activities (hours/week) |  |  | **0.04** |
| <1 | 127 (17.9) | 31 (29.8) |  |
| 1-2 | 350 (49.3) | 47 (45.2) |  |
| >2 hours/ week | 233 (32.8) | 26 (25.0) |  |
| Strenuous physical activities (hours/week) |  |  | 0.36 |
| <1 | 402 (56.6) | 66 (63.5) |  |
| 1-2 | 253 (35.7) | 34 (32.7) |  |
| >2 | 55 (7.7) | 4 (3.8) |  |

Supplementary table 7.2. Analyses of participant characteristics in the Safe Step intervention group based on whether the participants had downloaded the application or not.

S8. Adverse events during exercise with the Safe Step application

Encouragingly, a limited number of adverse events were reported during exercise sessions using the Safe Step application. Thirty-eight (4.7%) of the participants in the exercise group initially reported a fall during an exercise session with the Safe Step application. Twenty-two of these participants answered the follow-up e-mails and it was then revealed that only five of them had actually fallen during exercise using the Safe Step application; None of these five participants sustained an injury because of falling, but three of them experienced repeated falls during exercise.  This observation implies a potential misunderstanding among participants regarding the inquiries pertaining to falls during training, thereby initially resulting in an overestimation of falls and associated injuries linked to the training regimen.

Of the sixteen participants that did not respond to the follow-up email, five participants had reported injuries in the monthly fall report, including 1 with soft tissue injuries, and 4 with fractures that did not necessitate hospitalization (3 arm/wrist and 1 back). However, due to the potential misunderstanding described above, it is unclear whether these falls were actually related to a training with the Safe Step application.
